# Characterizing workplace exposures to nano-TiO_2_ in Singapore: potential risks and mitigation strategies

**DOI:** 10.1093/annweh/wxaf068

**Published:** 2025-11-06

**Authors:** Sriram Prasath, Kavitha Palaniappan, Sally Chan

**Affiliations:** School of Health Sciences, The University of Newcastle, Callahan, NSW 2308, Australia; School of Health Sciences, The University of Newcastle, Callahan, NSW 2308, Australia; Tung Wah College, 31 Wylie Road, Homantin, Hong Kong

**Keywords:** engineered nanomaterials (ENMs), nano safety, nano-TiO2, occupational exposure assessment, particle number concentration, regulatory frameworks, risk management

## Abstract

**Objectives:**

Engineered nanomaterials (ENMs), particularly nano-titanium dioxide (nano-TiO_2_), are widely used across industries in Singapore, raising concerns about potential worker exposure. This study aimed to quantify occupational exposures and emissions at workplaces handling nano-TiO_2_, assessing work practices, usage patterns and workplace controls.

**Methods:**

Occupational exposure to nano-TiO_2_ was assessed across 7 workplaces (laboratories, manufacturing, downstream application, and recycling). Methods for characterizing personal exposure included personal gravimetric sampling (NIOSH 0600), elemental analysis (NIOSH 7300), and scanning electron microscopy (SEM), while real-time particle number concentration (PNC) monitoring was done to understand the particle distribution in the workplace environment during the tasks performed. Workplace observations included measurement of dimensions of the work area, existing control measures (engineering, administrative, and personal protective equipment), nature of nano-TiO_2_ handling practices, forms, quantities, particle size, and state changes of the nano-TiO_2_ used.

**Results:**

Personal exposure samples were collected from 30 workers across workplaces. These include: 7 in laboratory, 10 in manufacturing, 6 in spraying, and 7 in shredding/recycling. Of these, 3 samples, collected during bulk loading and spraying activities, exceeded the NIOSH recommended exposure limit (REL) for ultrafine nano-TiO_2_ (0.3 mg/m^3^). Electron microscopy analysis of the samples exceeding the NIOSH REL for ultrafine nano-TiO_2_ during spraying revealed that the nano-TiO_2_ particles were predominantly in the size range of 80 to 147 nm. Respirable dust concentration and PNC were positively correlated for higher-risk activities, with peak PNC observed at the workplaces where spraying applications were performed.

**Conclusions:**

To our knowledge, this is the first study evaluating nano-TiO_2_ workplace exposure in Singapore. Exposure levels were generally low, likely due to prevalence of small-scale and research-based applications but varied significantly across workplaces for activities such as spraying, bulk loading and manufacturing. Singapore's current regulatory approach (TR 73) establishes exposure limits but lacks specific guidance on control measures. A more holistic regulatory framework is needed, providing tailored recommendations for diverse workplace exposure scenarios.

What's Important About This Paper? This study describes occupational exposures to nanoscale titanium dioxide across various workplaces in Singapore. Exposures showed variability across workplaces, but were generally within acceptable levels. The findings contribute to understanding of exposures with this material, and suggest the need for a holistic regulatory framework.

## Introduction

Engineered nanomaterials (ENMs) have revolutionized numerous industries, with titanium dioxide nanoparticles (nano-TiO_2_) gaining prominence in products ranging from paints and cosmetics to food additives and plastics. The global nano-TiO_2_ market reached 100,000 tons in 2020, with market value exceeding USD 22 billion in 2024. The market is expected to grow at a rate of 5.8% between 2024 and 2032 (Titanium Dioxide Nanomaterials Market Report. Global Market Insights 2024, 2025). This rapid proliferation, however, raises concerns about potential human exposure and associated risks, necessitating comprehensive research into the safety and impact of these materials (Semenzin et al. 2019). Studies indicate that inhaled nano-TiO_2_ particles can migrate beyond the respiratory system, reaching the brain and entering systemic circulation, potentially affecting organs such as the kidneys and liver (Shakeel et al. 2016). Research on occupational exposure has revealed that smaller particle sizes of anatase nano-TiO_2_ (10 to 30 nm) correlate with increased risk of inflammatory responses and oxidative stress among workers in production facilities (Higashikubo et al. 2021; Bonetta et al. 2022). Similarly, studies have identified that workers handling products containing nano-TiO_2_, such as cosmetics and clothing sales people, have shown elevated levels of oxidative stress markers (Lee et al. 2020).

Traditional mass-based metrics, long considered standard in occupational exposure assessment, have proven inadequate for capturing the potential risks associated with ENMs (Osunsanya et al. 2001). This inadequacy stems primarily from the high surface area-to-volume ratio and agglomeration potential characteristic of many ENMs, including nano-TiO_2_ (Lee et al. 2010). Alternative metrics, including number concentration, surface area, size distribution and other parameters, have been proposed in various studies to address these limitations (McGarry et al. 2016; Ding et al. 2017; Minelli et al. 2019; Oberbek et al. 2019). Multiple studies have corroborated the limitations of mass-based measurements for ENM exposure assessment, leading to the proposal of including particle number concentration (PNC) and Lung Deposited Surface Area (LDSA) as an alternative metric due to their advantages in real-time measurement capabilities and potential for assessing control efficacy (Fransman et al. 2008; Clark et al. 2012; Gordon et al. 2014; Simkó et al. 2014; McGarry et al. 2016). However, these alternative metrics are not without limitations. For instance, real-time instruments such as condensation particle counters (CPC) or diffusion chargers used for LDSA measurements are nonselective, sampling the total aerosol present rather than specifically isolating the ENM of interest. This lack of specificity can confound data interpretation, particularly in environments with mixed aerosols from uncontrolled sources (eg, background particles, incidental emissions), potentially overestimating exposure to the target ENMs (Dahm et al. 2013).

Despite these limitations, in occupational settings, mass concentration and number concentration have emerged as particularly valuable metrics for assessing exposure to ENMs due to their relative ease of measurement and the availability of established exposure limits for benchmarking. The National Institute for Occupational Safety and Health (NIOSH) has established mass concentration-based exposure limits, while the Dutch nano reference values (NRVs) provide number concentration benchmarks for various handling tasks (NIOSH, 2009, 2016, 2022; Broekhuizen and Hendrikx 2013). This standardized, task-based approach, particularly valuable for ENMs and its batch processing and daily variability, allows for harmonized data collection and comparison across different exposure scenarios (Brouwer et al. 2009; Savolainen et al. 2010).

Singapore, while not producing nano-TiO_2_, incorporates it across various sectors including research laboratories, electronics manufacturing, and construction materials. (Nair et al. 2011; Sng et al. 2011; Ting et al. 2015). Applications extend to Singapore's urban environment, aligning with smart city and sustainable building initiatives (Zhang et al. 2015; Chew et al. 2017) (Li et al. 2020). This widespread application necessitates comprehensive workplace exposure assessment to identify potential occupational exposure pathways and inform evidence-based controls. This study aims to quantify occupational exposure to nano-TiO_2_ across research laboratories, electronics manufacturing facilities, industrial powder processing plants, aerosol applications, and IT asset recycling sites. By analyzing exposure profiles and conducting comparative assessments across these workplace environments, the study will identify processes associated with elevated exposure risks and evaluate the effectiveness of existing control measures adopted at these workplaces for nano-TiO_2_ handling.

## Materials and methods

### Exposure assessment strategy

The exposure assessment strategy focused on characterizing exposure patterns across workplace categories with different nano-TiO_2_ handling activities. The different workplace categories are laboratory-scale powder handling, industrial-scale manufacturing and production processes, aerosol application, and material recycling operations. Due to fundamental differences in handling processes, material quantities, and physical forms of nano-TiO_2_ across these workplaces, the analysis focused on characterizing the range of exposures within each workplace category rather than assuming exposure similarity. Candidate workplaces were identified through a comprehensive outreach effort targeting all known nano-TiO_2_ handling facilities in Singapore. This involved contacting more than 40 companies directly, as well as targeted searches within online nanotechnology forums and industry conference proceedings. Relevant databases from associations such as SingNano—Singapore NanoTechnology Network and Asia Nano Forum—were utilized to obtain contact details of company representatives in Singapore. Snowballing methods were also employed by requesting association members to forward the study links or flyers to relevant industry contacts within their networks. Following the provision of an information statement, consent from both companies and employes was obtained before participation in the study.

The selection of workplaces was determined through formal invitations sent to nano-TiO_2_ handling facilities identified in Singapore's industrial registry. A cross-sectional survey was conducted among 65 companies in Singapore identified as potentially handling nanomaterials. The survey collected information on nano-TiO_2_ usage patterns, quantities handled, and potential for worker exposure (Prasath et al. 2025). Of these, 7 workplaces agreed to participate, representing various stages of product usage in Singapore. These facilities reflected a range of operational contexts and control strategies. These participating facilities constituted the complete set of companies willing to engage in our research. Among them, only 1 facility that used nano-TiO_2_ spraying technologies consented to assessment. Samples from these workplaces were collected exclusively during active TiO_2_ handling tasks (eg, weighing, spraying), with pumps operating continuously to capture cumulative task-based exposure, accounting for brief gaps within the tasks. Despite these participation constraints and variations in material quantities and physical forms between workplaces, the selected workplaces provided initial evaluation of nano-TiO_2_ handling scenarios in Singapore's industrial landscape.

### Measurement methods

The exposure assessment followed NIOSH-USA (NIOSH, 2009, 2022) protocol and additionally included PNC measurements. Respirable fraction samples were collected using SKC aluminum cyclones with 37-mm cassettes, using PVC filters for gravimetric analysis (NIOSH Method 0600) and mixed cellulose ester (MCE) filters for elemental titanium analysis (NIOSH Method 7300), attached to pre-calibrated SKC XR 5000 pumps with a flow rate of 2.0 litres per minute. Analysis for elemental titanium was performed at ALS laboratories and gravimetric analysis was performed at Institute of Occupational Medicine (IOM). Both the PVC and MCE filters used in this study were provided by their respective analyzing laboratories (ALS and IOM). A minimum of 3 personal exposure samples were collected per workplace during nano-TiO_2_ handling activities, totaling 30 samples across all workplaces for both respirable dust and elemental titania. One field blank sample was collected for each workplace. Following NIOSH protocol, samples exceeding 0.3 mg/m^3^ mass concentration underwent scanning electron microscopy (SEM) analysis to assess particle size and particle agglomeration. PNC was measured using a Condensation Particle Counter (CPC, model 3007, TSI Inc., USA) for 10 to 1,000 nm particles at 1-min intervals during working hours. Static monitoring was conducted with the CPC placed 1.5 m above ground within 0.5 to 1 m of the workplace activities. The CPC was strategically positioned near the workers under observation, while ensuring minimal disruption to their activities. To establish baseline levels, indoor background measurements (adjusted for background concentrations) were collected prior to the commencement of daily operations in each workplace. Due to logistical constraints and ongoing plant activities, pre-startup controls and post-task measurements in 4 of the 7 participating workplaces were conducted. The remaining 3 workplaces did not permit the use of the CPC because it failed to meet their intrinsic safety (IS) requirements.

Detailed observations were conducted at each of the 7 workplaces, documenting facility layouts, work processes, quantities of nano-TiO_2_ handled, engineering controls, and personal protective equipment (PPE) used. Respirable mass concentration, elemental titanium, and PNC were monitored at each workplace during a single day of operation, maintaining continuous measurements throughout work activities. These measurements, combined with our detailed observations of workplace practices and controls, provided crucial context for identifying potential exposure factors and interpreting the results.

### Description of workplaces

The nano-TiO_2_ exposure studies were conducted across 7 diverse workplaces (WP1 to WP7) in Singapore's industrial sector, representing the principal applications of nano-TiO_2_:

Laboratory settings (WP1-WP2):

- WP1: Research laboratory conducting flocculation studies, handling 50 to 200 g daily in slurry (liquid) form for a shift of 8 h.
- WP2: Pigment color testing laboratory, handling 800 g to 1 kg daily in powder form (initially, then converted to liquid with binder) for a shift of 8 h

Manufacturing facilities (WP3-WP4):

- WP3: Cleanroom facility with manual powder transfer from reactor, handling approximately 200 g daily (up to 1 kg normally) in powder form for a shift of 8 h.
- WP4: Industrial facility for plastic polymer production, handling 10 to 12 kg per batch operation in powder form for a production cycle of standard shift duration (6 to 8 h)

Application (WP5):

- WP5: Office setting implementing nano-TiO_2_ spraying for antimicrobial coating, handling 2.5L of suspended solution (containing approximately 800 g nano-TiO_2_) for an application session of standard application duration.

IT Asset Recycling (WP6-WP7):

- WP6: Data center with shredding operations for nano-TiO_2_ infused IT assets, processing 550 to 600 disks with embedded nano-TiO_2_ during a recycling shift of normal operational hours
- WP7: Secondary data center with shredding operations for nano-TiO_2_ infused IT assets, processing 205 disks (normally up to 550) with embedded nano-TiO_2_ during a recycling shift of normal operational hours.

**Fig. 1.**
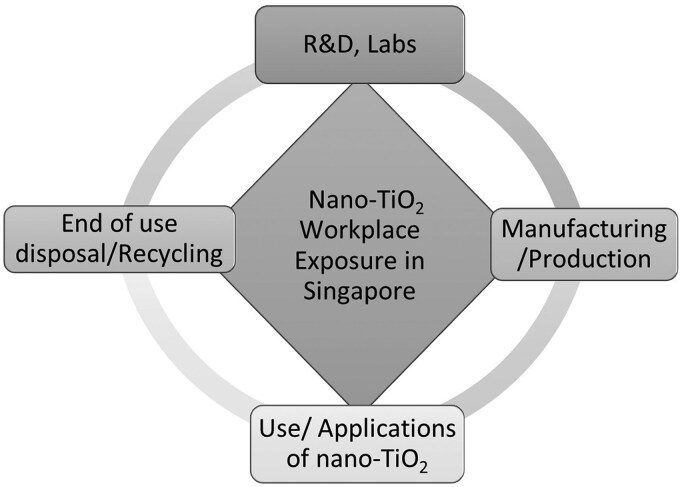
Nano-TiO_2_ exposure across workplaces in Singapore.

Personal exposure sampling was conducted throughout complete work shifts to capture time-weighted average exposures. Sampling duration varied by workplace categories: laboratory handling (4.0 to 6.4 h), manufacturing operations (4.0 to 5.9 h), spraying applications (3.0 to 3.1 h), and recycling activities (2.4 to 5.8 h). Figure 1 illustrates the exposures to Nano-TiO_2_ in Singapore.

### Statistical analysis

Statistical analysis was performed using Expostats (Lavoué et al. 2019), a web-based toolbox developed by the University of Montréal for interpreting industrial hygiene measurements under the lognormal distribution. Expostats was used to generate summary statistics across workplace categories. For measurements below the limit of detection (LOD), the LOD/2 substitution method was applied. Personal time-weighted average exposure measurements were directly compared to the NIOSH REL of 0.3 mg/m^3^ for ultrafine TiO_2_. Separately, PNC measurements were compared with the Dutch NRV of 40,000 particles/cm^3^.

## Results

### Personal exposure measurement results

The study, conducted from 2019 to 2022, across 7 workplaces (WP1 to WP7) represented different types of nano-TiO_2_ handling in Singapore. Table 1 summarizes exposure measurements across workplace categories with titanium concentrations and particle number concentrations where available.

**Table 1.** Summary statistics of nano-TiO_2_ exposure measurements by workplace type.

Workplace category	Workplaces	n	GM (mg/m^3^)	AM (mg/m^3^)	GSD	95th %ile (mg/m^3^)	PNC (particles/cm^3^, range)	Exceedances (NIOSH REL/Dutch NRV)	Comments
Laboratory settings	WP1–WP2	7	1.42 × 10^−4^	2.01 × 10^−4^	2.54	6.69 × 10^−4^	3,342 to 13,545	0/7; Below NRV	—
Manufacturing/production	WP3–WP4	10	5.06 × 10^−2^	1.79 × 10^−1^	14.5	2.57	222 to 9,930^a^	2/10; Below NRV in WP3; N/A WP4^a^	—
Spray Application	WP5	6	3.48 × 10^−3^	1.89 × 10^−1^	46.3	0.542	3,035 to 58,788	1/6; Above Dutch NRV	NRV exceedance during spraying activity.
End-of-use/recycling	WP6–WP7	7	5.28 × 10^−4^	6.25 × 10^−4^	1.8	1.53 × 10^−3^	Not measured	0/7; N/A	—

PNC, particle number concentration; GM, geometric mean; AM, arithmetic mean; GSD, geometric standard deviation.

All values below LOD estimated as LOD/2.

^a^PNC not measured at WP4.

Summary statistics showed that laboratory settings (WP1-WP2; *n* = 7) consistently had low titania exposures with a geometric mean of 1.42 × 10^−4^ mg/m^3^ and a variability (geometric standard deviation [GSD] = 2.54). Manufacturing facilities (WP3-WP4; *n* = 10) exhibited higher titanium exposures with a geometric mean of 5.06 × 10^−2^ mg/m^3^ and high variability (GSD = 14.5). This variability reflects fundamental differences between the open manufacturing environment (WP4) and controlled cleanroom environment (WP3). Spraying operations (WP5; *n* = 6) showed the highest variability (GSD = 46.3) with a geometric mean of 3.48 × 10^−3^ mg/m^3^ and exposures ranging up to 0.88 mg/m^3^, exceeding the NIOSH REL of 0.3 mg/m^3^. Recycling operations (WP6-WP7; *n* = 7) demonstrated low variability (GSD = 1.8) with a geometric mean of 5.28 × 10^−4^ mg/m^3^ and consistently low exposures. Respirable dust concentrations remained below the detection limit (0.030 mg/m^3^) at most workplaces. When detectable concentrations were observed at WP3, WP4, and WP5, all measurements were below the occupational exposure limit of 3 mg/m^3^. Given that respirable dust measurements were predominantly below detection limits and showed no exceedances of occupational limits, subsequent analysis focused on nano-TiO_2_-specific metrics (titanium concentration and PNC) which provided more relevant exposure characterization.

PNC measurements were obtained at 4 workplaces (WP1, WP2, WP3, WP5) with background correction applied is presented in Table 2. Laboratory settings (WP1 and WP2) and the cleanroom environment (WP3) maintained low particle levels, consistently below 10,000 particles/cm^3^. In contrast, WP5 demonstrated significantly higher particle concentrations during spraying activities and was above the Dutch NRV for TiO_2_ of 40,000 particles/cm^3^. The temporal profile at WP5 followed a distinct pattern: particle numbers increased with spraying initiation, reached a plateau during operations, and declined as activities ceased.

**Table 2.**
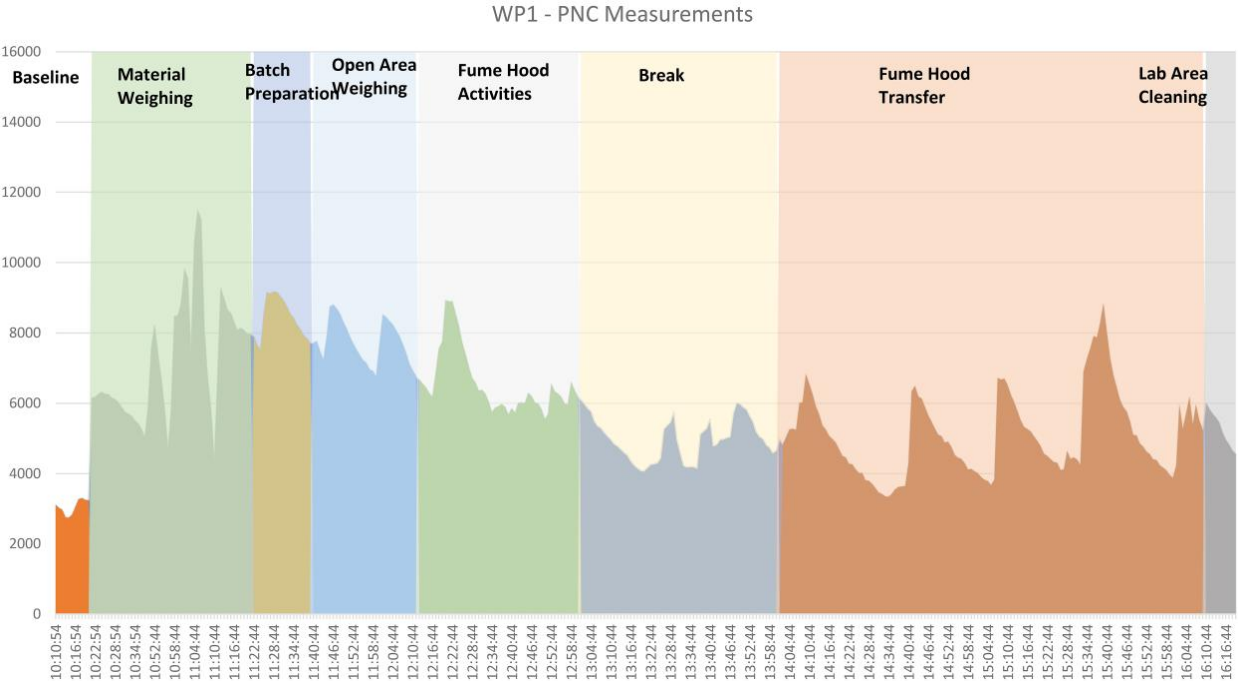

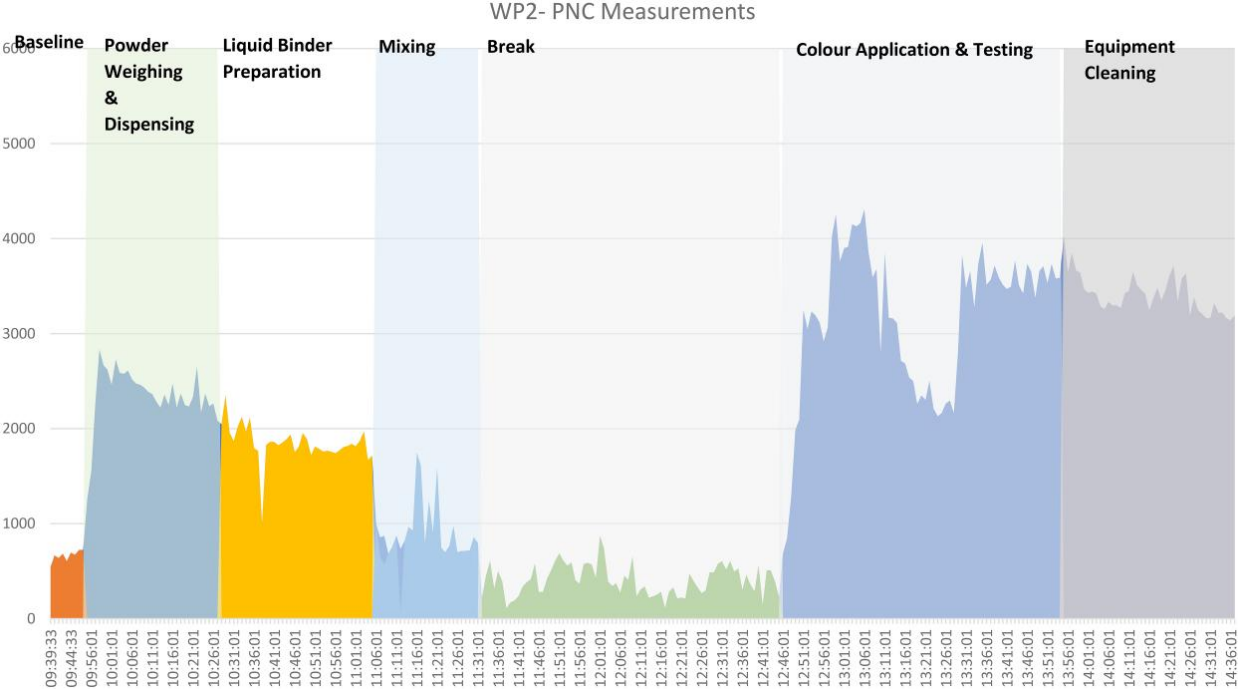

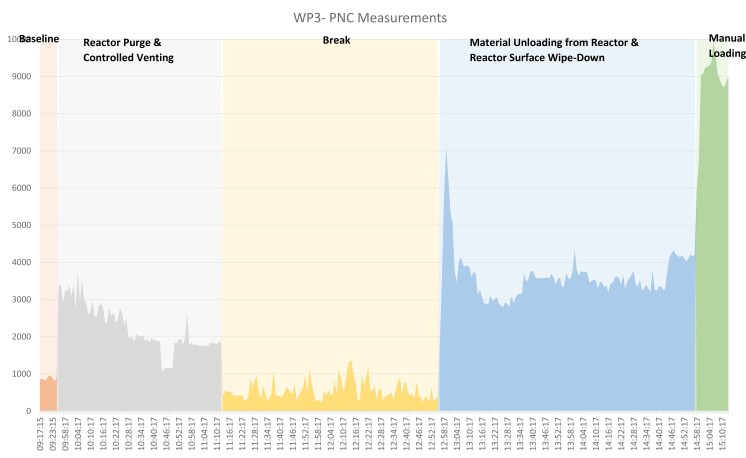

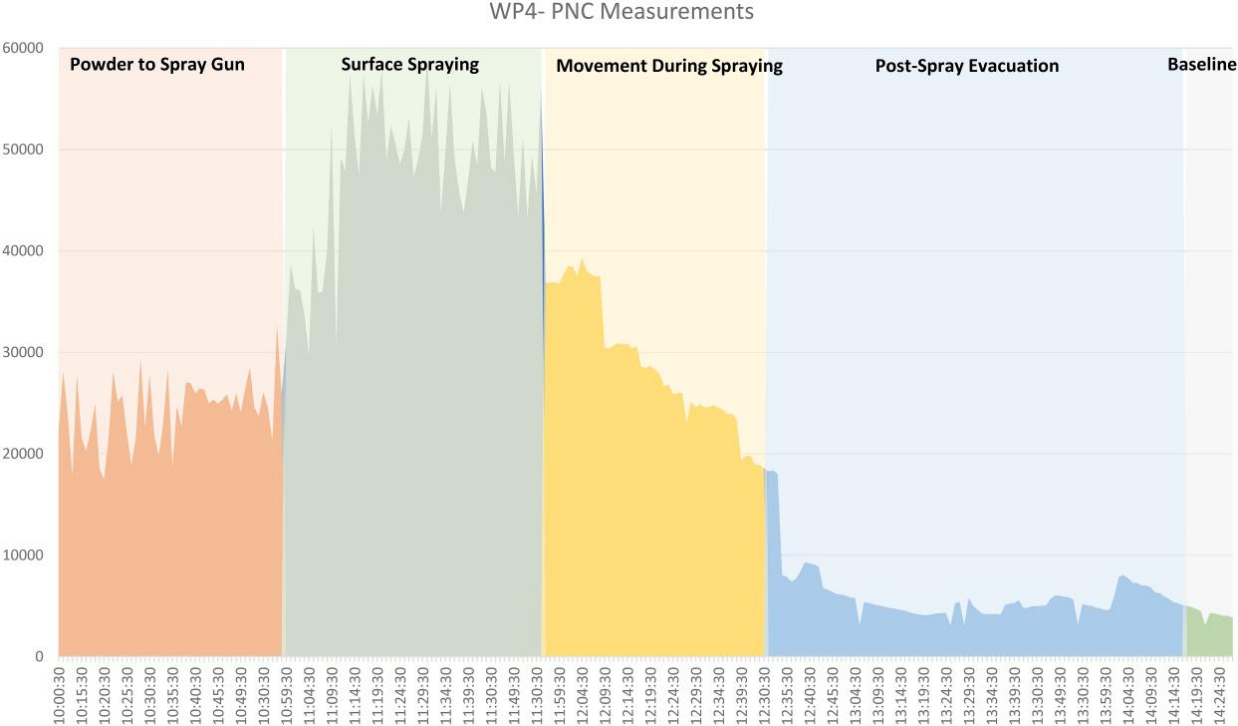
PNC temporal variation pattern across workplaces.

**Fig. 2.**
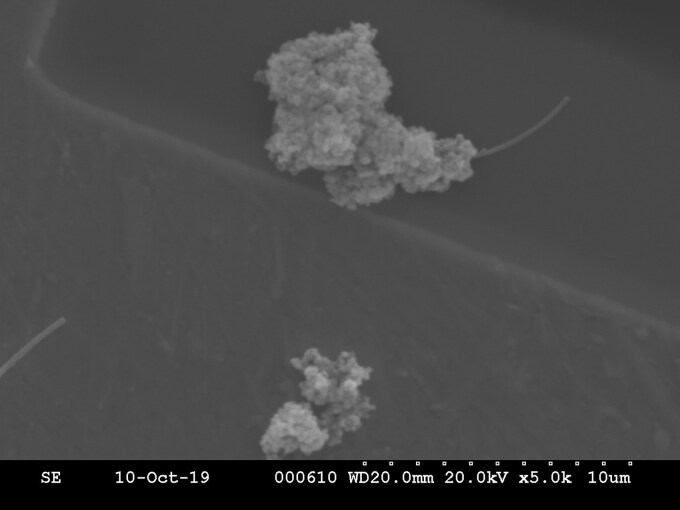
SEM image of agglomerated particles in WP5.

**Fig. 3.**
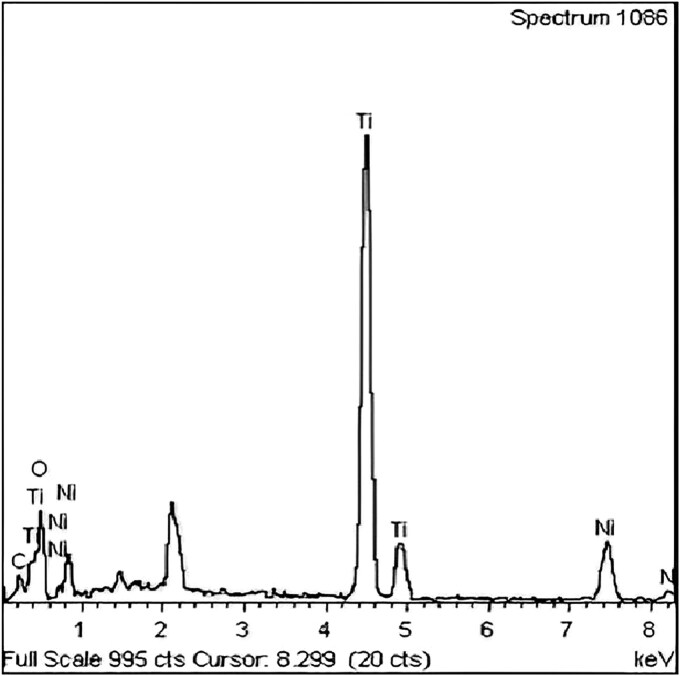
EDXS spectrum image of nano-TiO_2_ in WP5.

SEM analysis at WP5 revealed particle agglomeration with 2 distinct morphologies: minimally agglomerated and extensively agglomerated structures. The SEM image indicated that airborne nanoparticles formed agglomerates in the range of 80 to 147 nm Fig. 2. Energy dispersive X-ray spectroscopy (EDXS) spectrum of nano-TiO_2_ particles at WP5 is shown in Fig. 3. SEM analysis of WP4 samples could not be performed due to sample degradation during storage. Details of engineering controls, administrative protocols, and PPE implemented at each workplace are summarized in Table 3.

**Table 3.** Activities, quantity, particle size, and other contextual information on controls.

Task category	Activity	Form of nano-TiO_2_ handled	Quantity of material handled on the day^a^	Quantity handled under normal use^a^	Size of nano-TiO_2_^a^ (nm)	Change of state of nano-TiO_2_	Existing controls during activity	Engineering controls	Administrative controls	PPE	Room dimension, LxWxH = V (m^3^)	No. of doors + Windows^b^
*Lab Handling (WP1)*	Nano-TiO_2_ weighed and handled in fume hood	Initial slurry of nano-TiO_2_ in suspended form. After drying, TiO_2_ weighed in powder form.	50 gm	200 grams	20 to 30	Slurry to free powder	Fumehood—during opening of the nano-TiO_2_ and no control during weighing on benchtop.	Fume hood	Trained to handle nano-TiO_2_ in fume hood	NA; PPE was not worn.	84	1 + 0
*Lab Handling (WP2)*	Nano-TiO_2_ weighed and handled in fume hood	Powder form initially handled. After binder added, material converted to liquid.	800 grams	800 gm to 1 kg	<100	Direct powder loading	Samples were weighed and handled in fume hood.	Fume hood	Trained to handle nano-TiO_2_ in fume hood	Half face respirator with P100 cartridge	201.6	1 + 0
*Manual Transfer (WP3)*	Nano-TiO_2_ removed from reactor in a cleanroom	Powder form	200 gm	1 kg	<100	Direct powder loading	Open area handling where powder removal is done from reactor to bag.	Custom capture hood on top of reactor.	Standard Operating Procedure	Powered Air Purifying Respirator with Hood (PAPR)	336	2 + 0
*Manual Charging (WP4)*	Charging of nano TiO_2_	Powder form	12 kg	10 to 12 kg	80 to 400	Direct powder loading	Open handling in production area	Flexible arm LEV	Standard Operating Procedure	Half face respirator with P100 cartridge	1,140	2 + 3
*Application—Spraying (WP5)*	Aerosolised nano TiO_2_ sprayed in office	Nano TiO_2_ in suspended form and aerosolised upon pressure	2.5L	800 gm	50 to 100	Liquid to aerosol	Open handling in open area	No	Trained to move away from the area immediately after spraying and wait for 2 hours to resume spraying activities	Disposable half face respirators	302.4	1 + 2^c^
*Disposal (WP6)*	Shredding of nano TiO_2_ infused IT assets	Nano-TiO_2_ embedded in solid form	550 to 600 disks	550 disks	65	Particle aerosolization due to shredding	Fully enclosed process of disposing the printed circuit boards in a machine, shredded material into recycle bin.	Enclosed process—shredder contained LEV	Restricting quantity to shred based on manufacturers recommendation	Half face respirator with P100 cartridge	84	1 + 0
*Disposal (WP7)*	Shredding of nano TiO_2_ infused IT assets	Nano-TiO_2_ embedded in solid form	205 disks	550 disks	65	Particle aerosolization due to shredding	Fully enclosed process of disposing the printed circuit boards in a machine, shredded material into recycle bin.	Enclosed process -shredder contained LEV	Restricting quantity to shred based on manufacturers recommendation	Half face respirator with P100 cartridge	70	1 + 0

^a^Information was obtained from each of the workplaces.

^b^The access points remained closed during work activities, and door/window opening frequencies were not monitored during sampling periods.

^c^The doors and windows remained opened during the spraying activity.

## Discussion

This study evaluated occupational nano-TiO_2_ exposures across 7 workplace settings in Singapore. Three personal exposure measurements exceeded the NIOSH REL of 0.3 mg/m^3^ for ultrafine TiO_2_, indicating elevated exposure risks in manufacturing and spraying operations.

Our findings are consistent with previous research on nano-TiO_2_ exposures across different workplace environments. Bressot et al. (2018) evaluated nano-TiO_2_ PNC in both laboratory-scale operations and spray applications, providing a valuable comparison to our study. Their laboratory measurements during TiO_2_ handling (Process Line 4) showed particle counts ranging from 2,500 to 5,000 particles/cm^3^, indicating minimal emissions during most handling operations. This aligns with our laboratory findings (WP1 and WP2) where mean concentrations remained consistently low below 10,000 particles/cm^3^. The spray application of nano-TiO_2_ in our study (WP5) had a mean PNC of 15,941 particles/cm^3^, compared to Bressot et al.'s spray applications, which showed mean TiO_2_ concentrations of 22,000 particles/cm^3^.

Another similar multi-workplace study by Ham et al. (2015) provides further context through their comprehensive comparison of workplace types, including 2 specific nano-TiO_2_ handling environments relevant to the present study. Their evaluation included Workplace A, which involved nano-TiO_2_ transferred to crucibles from storage with a general ventilation fume hood in a relatively small lab (120 m^2^), and Workplace D, which involved considerable TiO_2_ production in a larger facility (1,400 m^2^). These workplaces represent both laboratory-scale and industrial-scale TiO_2_ handling environments, similar to WP1 and WP4 in our study. Ham et al. found mean PNC of 8,458 particles/cm^3^ in laboratory settings and 19,612 particles/cm^3^ in manufacturing settings, showing higher concentrations in manufacturing facilities consistent with our observations across workplace categories. These comparative studies highlight the value of multi-metric approaches in exposure assessment. Mass concentration measurements successfully quantified high-exposure scenarios, while particle number concentrations detected emissions in lower-exposure activities where mass concentrations were below detection limits.

Exposure levels varied both within workplace categories and between workers performing similar tasks. WP5 operations showed high inter-worker variability (GSD = 46.3), while manufacturing processes differed between WP4 and WP3 (GSD = 14.5). WP3 operations illustrate that mass concentrations can remain low even when particle number concentrations vary during material handling. These observations highlight the need for multi-metric, activity specific assessment framework for ENM operations (McGarry et al. 2016; Asbach et al. 2017).

Particle characterization analysis provided additional insights. SEM analysis revealed that airborne particles at WP5 were agglomerated rather than present as individual particles, underscoring the role of particle form in exposure evaluation. Ham et al. (2015) emphasized the need to distinguish between individual particles and agglomerates/aggregates, as particle behavior influences both exposure potential and toxicological relevance foe ENMs.

Extending prior research (Bressot et al. 2018), our study combined mass concentration and particle number measurements with direct comparison to regulatory benchmarks (NIOSH REL of 0.3 mg/m^3^; Dutch NRV of 40,000 particles/cm^3^), providing a more complete assessment than single-metric approaches. This multi-metric assessment allowed comparisons across workplace scenarios and showed that current guidance values may not capture the full range of exposure variability. ENM exposures were activity dependent, with critical tasks such as material transfer or spraying contributing disproportionately to overall exposure, highlighting the importance of activity-specific evaluation when designing control strategies.

Control measure implementation varied across workplace settings. Both manufacturing facilities (WP3 and WP4) used local exhaust ventilation, but WP4 showed higher exposures due to open handling processes compared to WP3's enclosed cleanroom environment. This aligns with previous findings that process-specific factors significantly influence control effectiveness (Schmid et al. 2010; Heitbrink et al. 2015). These variations emphasize the importance of activity-specific engineering controls rather than relying solely on standardized control specifications, particularly in facilities handling ENMs with varying sizes, physical forms and quantities. Administrative control implementation also varied across workplaces, with inconsistent compliance observed in post-application evacuation protocols, consistent with previous research highlighting challenges in maintaining administrative controls in industrial settings (Health 2011; Clark et al. 2012). Together, these findings suggest that ENM exposure controls require activity-specific implementation and effectiveness evaluation.

### Limitations

A major limitation in this study was the difficulty in recruiting companies, despite using personalized emails, snowballing, and event flyer distribution. Response rates remained low, further reduced by the COVID-19 pandemic, which restricted visitor access for research. Concerns about regulatory consequences of disclosing nanomaterial handling practices likely contributed to low participation, despite assurances of confidentiality.

High within-category exposure variability (GSDs of 14.5 for manufacturing and 46.3 for spraying) indicates that workplace categories do not represent homogeneous exposure conditions. Facilities within the same industry sectors showed diverse operational characteristics and control implementations. This diversity limits the applicability of traditional SEG approaches for ENM exposure assessment.

Technical constraints affected data collection, as the CPC was excluded from 3 workplaces (WP4, WP6, and WP7) due to noncompliance with site mandated IS requirements. Additionally, SEM characterization of WP4 samples could not be performed due to sample degradation during storage during COVID period. Single-day sampling at each workplace may not capture temporal variations in exposure patterns that could occur across different operational periods or seasonal conditions.

## Conclusion

This research demonstrates that conventional approaches to ENM exposure assessment require refinement for emerging workplace scenarios. The substantial exposure variability observed across workplace categories (GSDs ranging from 1.8 to 46.3) challenges assumptions about industry-based exposure prediction and supports the need for activity-specific assessment strategies rather than broad workplace categorization approaches. Furthermore, our multi-metric approach reveals that relying on single exposure metrics can significantly underestimate risk in certain scenarios while potentially overestimating it in others. These findings have practical implications for developing more effective exposure control strategies and evidence-based approaches to ENM workplace safety.

Future research should expand these methods to additional ENM types and workplace scenarios to develop comprehensive exposure assessment frameworks for the nanotechnology industry. Longitudinal studies examining the consistency of exposure patterns over time and the long-term effectiveness of implemented control measures would further strengthen these evidence-based approaches.

## Data Availability

The data that support the findings of this study are available from the corresponding author upon reasonable request.
